# Mesoscale eddies influence the movements of mature female white sharks in the Gulf Stream and Sargasso Sea

**DOI:** 10.1038/s41598-018-25565-8

**Published:** 2018-05-09

**Authors:** Peter Gaube, Camrin D. Braun, Gareth L. Lawson, Dennis J. McGillicuddy, Alice Della Penna, Gregory B. Skomal, Chris Fischer, Simon R. Thorrold

**Affiliations:** 1 Applied Physics Laboratory, University of Washington, Seattle, Washington, USA; 2 Massachusetts Institute of Technology-Woods Hole Oceanographic Institution Joint Program in Oceanography/Applied Ocean Science and Engineering, Cambridge, Massachusetts, USA; 3 Woods Hole Oceanographic Institution, Woods Hole, Massachusetts, USA; 4 Division of Marine Fisheries, New Bedford, Massachusetts, USA; 5 OCEARCH, Park City, Utah USA

## Abstract

Satellite-tracking of mature white sharks (*Carcharodon carcharias*) has revealed open-ocean movements spanning months and covering tens of thousands of kilometers. But how are the energetic demands of these active apex predators met as they leave coastal areas with relatively high prey abundance to swim across the open ocean through waters often characterized as biological deserts? Here we investigate mesoscale oceanographic variability encountered by two white sharks as they moved through the Gulf Stream region and Sargasso Sea in the North Atlantic Ocean. In the vicinity of the Gulf Stream, the two mature female white sharks exhibited extensive use of the interiors of clockwise-rotating anticyclonic eddies, characterized by positive (warm) temperature anomalies. One tagged white shark was also equipped with an archival tag that indicated this individual made frequent dives to nearly 1,000 m in anticyclones, where it was presumably foraging on mesopelagic prey. We propose that warm temperature anomalies in anticyclones make prey more accessible and energetically profitable to adult white sharks in the Gulf Stream region by reducing the physiological costs of thermoregulation in cold water. The results presented here provide valuable new insight into open ocean habitat use by mature, female white sharks that may be applicable to other large pelagic predators.

## Introduction

The open ocean represents the largest ecosystem on earth and is responsible for approximately half of the planet’s net primary production^1,2^. This pelagic ecosystem is structured by mesoscale eddies^3^ and fronts^4^. Mesoscale eddies in the Northwest Atlantic are counted amongst some of the most energetic ocean features^5^, fueling vertical nutrient fluxes^6^ while trapping and transporting entire biological communities over vast distances^7^. In the North Atlantic, cyclonic eddies are generally characterized by elevated near-surface chlorophyll (a proxy for phytoplankton biomass); while anticyclonic eddies are predominantly associated with low surface chlorophyll^7^, they can also exhibit high surface chlorophyll^8^. Despite high variability in phytoplankton biomass associated with these eddies, the sub-tropical North Atlantic is often characterized as an ocean desert that may have increased in size over the past 15 years^9^. Yet this region hosts populations of large pelagic fishes, including commercially important tunas^10^, billfish^11^ and sharks^12^. It has proved difficult to reconcile the apparent contradiction of large populations of apex predators residing in barren ocean waters. However, resolving this paradox is critical if food web models for the open ocean are to provide guidance for ecosystem-based management of pelagic systems^13^.

The proliferation of satellite tracking studies has significantly increased our understanding of the movements of apex predators in the ocean^14–16^. Results from tag deployments have, for instance, shown basin-scale movements of large pelagic fishes over months to years^15^ and revealed diving behaviors that span from surface waters to the deep ocean^17^. However, the light-level geolocation method used by traditional pop-up satellite archival transmitting (PSAT) tags to determine position is not sufficiently accurate (ca. 50–100 km) to quantitatively link animal movements with specific mesoscale oceanographic features^18^. Yet these features are likely to have profound effects on ocean ecology if large pelagic fishes are able to detect the presence of favorable conditions associated with mesoscale eddies and to adjust their horizontal and vertical movements to increase interactions with prey, as has been shown for marine mammals^19^, seabirds^20^, and turtles^21,22^. However, relatively little is known about collocation of pelagic fish movements with mesoscale oceanographic features. Here, we use data from deployments of two types of satellite tags to reconstruct the movements of mature female white sharks (*Carcharodon carcharias*) and test the ability of the individuals to orient themselves with respect to mesoscale eddies and meanders in the Northwest Atlantic Ocean.

## Three-dimensional Movement

**Figure 1:**
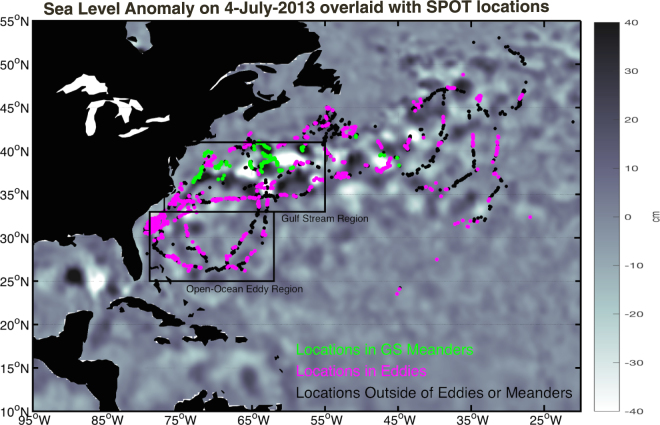
Map of the sea level anomaly on 4-July-2013 (shading) overlaid with SPOT tag positions of the 2 white sharks analyzed here. Positions inside eddies are shown as magenta points, in Gulf Stream meanders as green points, and outside of mesoscale features as gray points. The approximate bounds of the two study regions described in the text are indicated by the black boxes. This map was generated using the m_map toolbox (https://www.eoas.ubc.ca/~rich/map.html) implemented in Matlab R2017a.

We tracked the movements of two mature female white sharks with “Smart” Position or Temperature Transmitting (SPOT) tags mounted to the dorsal fin. The tags acquire position from an Argos satellite when the tag antenna is exposed to air at the surface and report data back via the same satellite system. Mean geolocation error of the SPOT tags was <5 km (see Methods section). Shark movements were collocated to the positions of mesoscale eddies and meanders identified and tracked in contemporaneous maps of sea surface height (SSH) constructed from satellite observations of sea level anomaly (SLA) mapped at ¼ degree horizontal grid resolution. The tagged white sharks showed a clear affinity for mesoscale oceanographic features, with 45% of all satellite-based positions inside westward propagating eddies that encompassed approximately 30% of the surface in the North Atlantic during tag deployment. When limiting the analysis to the region west of 55 W with the highest density of SPOT data and largest amplitude eddies (Fig. 1), the pattern was more pronounced, with 76% of all SPOT positions inside of eddies that in turn encompassed approximately 35% of the study region. These results indicate white sharks were either preferentially occupying, or retained within, the interiors of mesoscale eddies.

Comparison of observed movements to null, random-walk trajectories indicated that the two study animals were 20% more likely to be in the interior of anticyclones when compared to cyclones, revealing a partitioning of white shark locations by eddy polarity (Supplementary Fig. 1 and see Supplemental Information). The white sharks were also significantly more likely to be found in the cores compared to the periphery of anticyclones, and more likely to be found in the cores of anticyclones compared to the cores of cyclones (Supplementary Fig. 1). There was, however, no significant difference between the number of shark locations in the core compared to the periphery of cyclones. In summary, the white sharks frequented anticyclonic eddies more than cyclonic eddies, and were more commonly associated with the interiors of anticyclones than the periphery of these same type of eddies.

**Figure 2:**
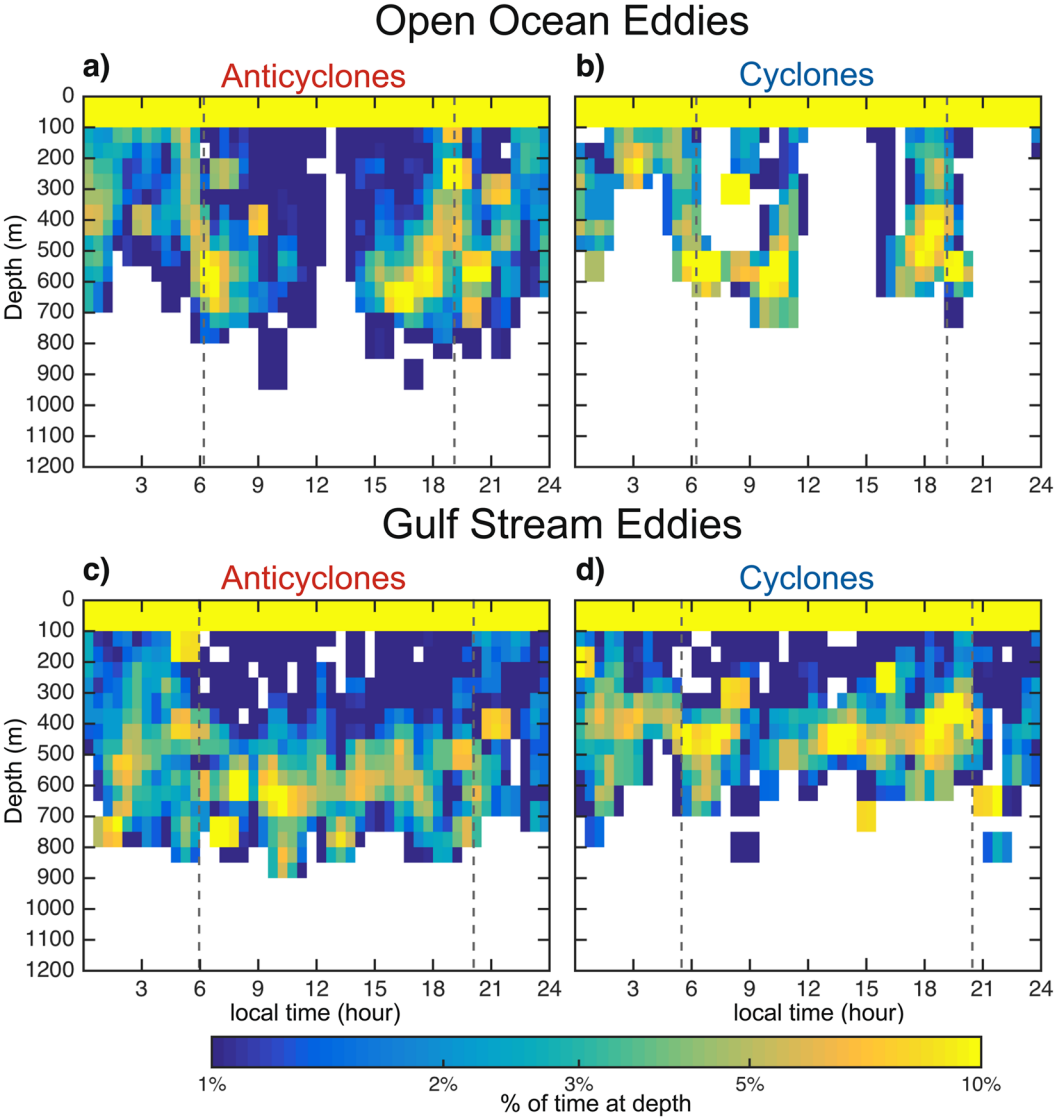
Two-dimensional histograms of dive depth as a function of local time. Dives within anticyclonic and cyclonic mesoscale eddies are shown in the left and right columns, respectively, on a log scale. Thin vertical broken grey lines represent the mean time of local sunrise and sunset. Dives occurring while the shark interacted with eddies in the open ocean region are shown in the top row and with eddies in the Gulf Stream region are shown in the bottom row. White indicates no data.

**Figure 3:**
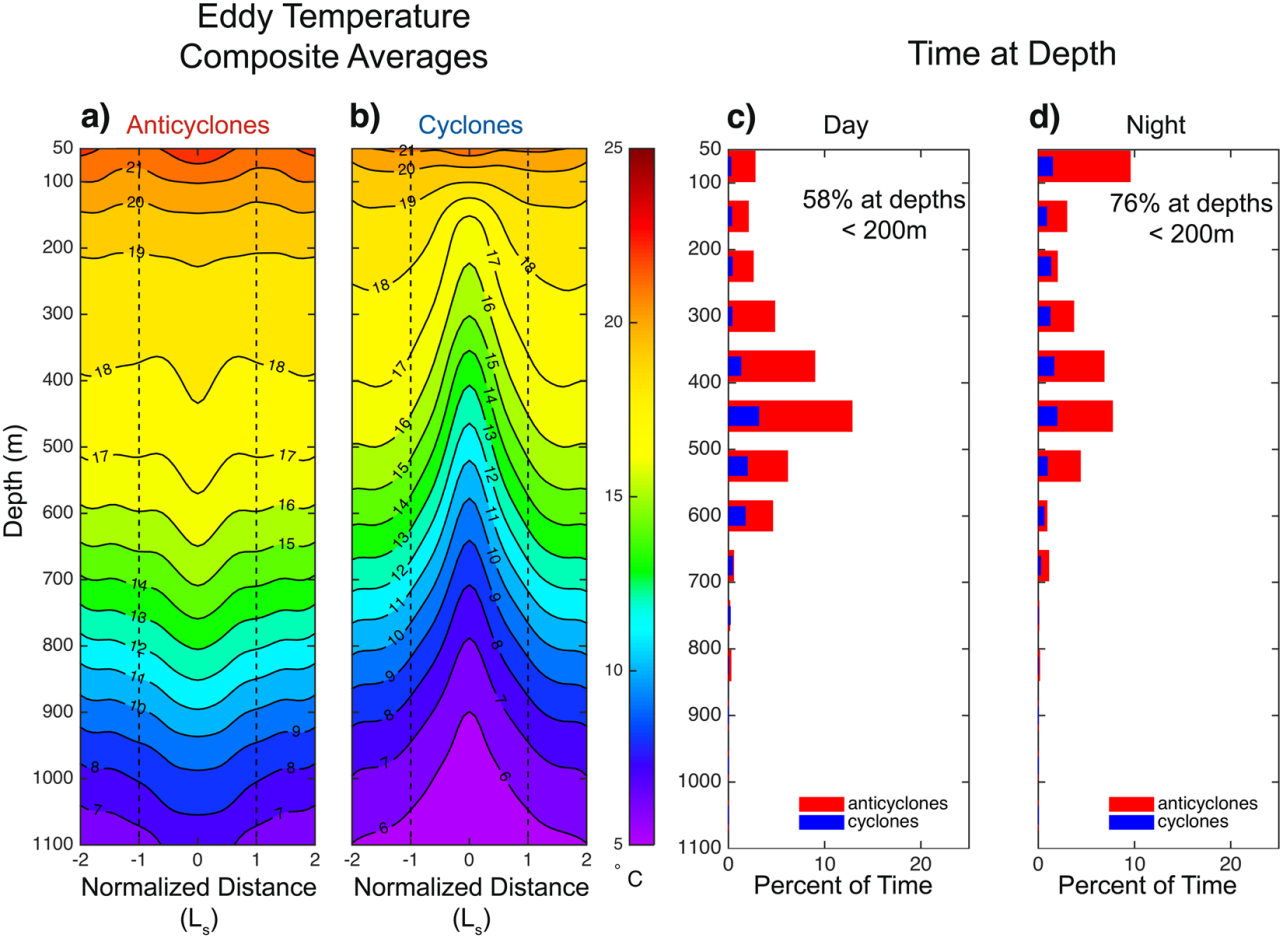
Composite averages of potential temperature from Argo floats in Gulf Stream (**a**) anticyclones and (**b**) cyclones. The time spent at depth computed from the 5-minute resolution dive data (see methods) in Gulf Stream anticyclones (red) and cyclones (blue) is shown as stepped lines during the (**c**) day and (**d**) night.

We reconstructed vertical movements of one of the tagged white sharks fitted with a pop-up satellite archival transmitting (PSAT) tag that logged temperature and depth at regular 5-min intervals during a six-month deployment. The locations of individual dives were determined by matching the time of the dive with the corresponding track chronology estimated from the SPOT positions. While in the open ocean (Fig. 1), the shark made frequent forays into the mesopelagic zone (200–1,000 m), primarily around the time of sunrise and sunset (Fig. 2a,b). When limiting the analysis to only those dives that reached a depth of at least 200 m, defined here as deep dives, we observed more than three times as many dives in open ocean anticyclones when compared to cyclones (Supplementary Fig. 2). The depth and duration of these deep dives were not, however, significantly different between eddies of opposite polarity in this region (Supplementary Fig. 2). In the Gulf Stream region (Fig. 1), deep dives into anticyclones were 57% more common than into cyclones (Supplementary Fig. 2). These dives were also significantly longer in duration and marginally deeper in anticyclones compared to cyclones (Supplementary Fig. 2). In these energetic Gulf Stream eddies, dive depths clustered around 400–600 m in anticyclones (Figs 2c and 3c,d) and 300–500 m in cyclones (Figs 2d and 3c,d). Deep dives occurred throughout the day and not primarily around sunrise and sunset, as was the case in the open ocean eddies (Fig. 2a and b). Furthermore, this individual spent more time at depth in Gulf Stream anticyclonic eddies compared to Gulf Stream cyclones and anticyclonic open ocean eddies. This differential use of the mesopelagic was more pronounced during daytime when this shark spent 50% of its time at depths >200 m within Gulf Stream anticyclonic eddies (Supplementary Table 1).

### Temperature Constraints on Diving Behavior

Temperature constraints on the vertical movements of white sharks may provide an explanation for the differences in dive behavior between regions and eddy types. To test this hypothesis, we extracted temperature-depth profiles from Argo floats reporting from the Gulf Stream region from 2002 through 2014 (see Methods for details). Composite average temperature profiles of eddies in this region indicated that anticyclonic eddies were characterized by displacement of isotherms downward by approximately 50 m (Fig. 3a). Downward displacement of isotherms resulted in moderate temperature anomalies of ~2.5 °C at a depth of 700–800 m (Supplementary Fig. 3k). Argo float profiles also revealed that isotherms in cyclonic eddies in this region were, on average, over 200 m shallower than outside of cyclones (Fig. 3b) resulting in large negative temperature anomalies with a maximum magnitude of −4.6 °C at 500 m (Supplementary Fig. 3l). The isotherm displacements of cyclones and anticyclones in the region examined here were, however, not symmetrical. The asymmetry resulted from the geometry of the domain in which a number of large amplitude cyclonic “cold-core rings” (or eddies) that originated from the Gulf Stream were included in the composites whereas the anticyclones were dominated by smaller amplitude eddies of open-ocean origin. Nonetheless, the warm interiors of anticyclonic eddies in this region may allow for longer deep dives than in comparatively colder water found in the interiors of cyclones as a result of thermal regulation requirements. Indeed, we observed that when the double-tagged white shark was in relatively cold cyclonic eddies, it was 20% and 42% more likely to be at the surface during the day and night (respectively) than at depth, when compared to the warmer anticyclonic eddies (Supplementary Table 1). Further evidence for the temperature regulation hypothesis came from the lack of a significant difference in either dive depth or duration as a function of polarity in open ocean eddies. There were smaller isotherm displacements associated with open ocean eddies compared to larger amplitude eddies in the Gulf Stream region (Supplementary Fig. 3), and, therefore, the thermal environments in both types of eddies were more similar. Thus, the lack of significant differences in the dive profiles between anticyclonic and cyclonic eddies in the open ocean region (Supplementary Fig. 2) is consistent with the temperature hypothesis.

The association of white sharks with anticyclones is perhaps counterintuitive because clockwise rotating anticyclonic eddies in the North Atlantic are often associated with low surface chlorophyll^7^, whereas cyclones have been observed to enhance chlorophyll and phytoplankton biomass^23^. However, some anticyclones in this region, in particular those containing an intra-thermocline lens of water, have been observed to contain large concentrations of diatoms in their cores resulting in enhanced primary production rates^24^. Moreover, deeper mixed layers in anticyclonic eddies can lead to enhanced chlorophyll in oligotrophic regimes^8^. Vertical and horizontal distributions of prey populations also likely influence the dive profiles of pelagic predators in mesoscale eddies, as white sharks are known to forage in the mesopelagic^25^. An acoustic survey across the North Atlantic, from Scotland to Nova Scotia, found that acoustic backscatter - a proxy for community biomass - in the mesopelagic zone was elevated in anticyclones compared to cyclones^26^. White sharks may, therefore, be accessing prey more efficiently in warm-core anticyclones. However, without direct observations of the distributions of mesopelagic organisms in the eddies frequented by the double-tagged individual in our study, we cannot diagnose the influence of eddy type on mesopelagic prey biomass, and thus on the diving behavior of the double-tagged white shark analyzed here. Nonetheless the double-tagged shark spent more time near the surface during the night when compared to the day (Fig. 3c,d and Supplementary Table 1), thereby providing circumstantial evidence that it was foraging on prey following a diel vertical migration pattern while within eddies.

### Conclusions

Several conclusions may be drawn from the comparison of this white shark’s diving behavior to the thermal structure of eddies in the Northwest Atlantic. First, the double-tagged white shark spent substantially more time in the mesopelagic in anticyclones, often diving to a depth corresponding to maximum acoustic backscatter reported in the earlier acoustics study of mesopelagic fishes in anticyclones versus cyclones^26^. Second, dives were of shorter duration in cyclonic eddies when compared to dives in anticyclones (Supplementary Fig. 2), suggesting that this white shark may have been foraging in the cyclones, but may have experienced thermal constraints that limited time spent at these depths when compared to anticyclones. Third, the diving behavior of the double-tagged white shark showed strong diel variability (Fig. 3c,d and Supplementary Table 1), consistent with the behavior of vertically-migrating prey^27^ and the day-night shift in vertical distribution of backscatter in the Northwest Atlantic^26^. Indeed, the tagged shark spent more than 76% of its time in the epipelagic, defined here as depth <200 m, during the night, compared to 58% during the day (Fig. 3c and d). Most recorded deep dives occurred during the day (Supplementary Table 1), when vertically migrating organisms are generally found in the mesopelagic.

Our study highlights the potential influence of mesoscale oceanographic features on the behavioral ecology of top predators. Our results suggest that warm sub-surface temperature anomalies in anticyclones may confer an energetic advantage for pelagic predators that are able to detect these features (*e*.*g*. physical and/or biological anomalies such as temperature or mesopelagic community changes) and then forage in the interiors of the eddies. The regular forays into the mesopelagic observed in the three-dimensional trajectories of the double-tagged white shark also suggest that mesoscale eddies, and the communities they support, may represent important habitat for some species of pelagic sharks. Yet we know remarkably little about the biology of this zone in the open ocean^28^. For instance, recent studies have suggested that the global biomass of fishes in the mesopelagic zone may have been underestimated by at least an order of magnitude^29^, suggesting that the mesopelagic zone might be a crucial foraging habitat for large pelagic predators. The observation of high biomass at mesopelagic depths in the pelagic ocean has generated interest from commercial fishing operations and raised the possibility of significant resource extraction from mesopelagic communities in the near future^13^. While we are still far from a complete understanding of the likely effects of this extraction on pelagic food webs, our results show that white sharks could be potentially impacted along with other pelagic predators such as swordfish^11^, bigeye tuna^27^, mobulid rays^17^, and beaked whales^30^.

Identifying the importance of mesoscale features to top predators provides the opportunity for adaptive spatial management strategies including dynamic Marine Protected Areas (MPAs) focused on important spawning or foraging habitats^31^. Limiting interactions between vulnerable or endangered species and fishing activity may also be facilitated by a better understanding of habitat use by pelagic predators in the open ocean^12^. Further understanding of the diets of top predators, coupled with knowledge of the mesoscale and submesoscale distribution of their prey field, needs to be obtained before effective spatial or ecosystem management options can be considered in the open ocean.

## Methods

### Eddy and Meander Identification and Tracking

In this study, mesoscale eddies and Gulf Stream meanders, defined here as coherent mesoscale structures (CMS), have been identified and tracked based on their signatures in SSH^5^. The altimeter-tracked eddy dataset used in this analysis is available online at http://cioss.coas.oregonstate.edu/eddies. Prior to CMS tracking, AVISO SLA fields were high-pass filtered in space to remove the effects of seasonal heating and cooling, resulting in daily SSH fields^5^ defined as:

1 $$\begin{array}{c}SSH=SLA-\langle SLA\rangle \end{array}$$

where <SLA> denotes the low-pass filtered AVISO SLA fields smoothed using a LOESS filter with a half-power cutoff of 20° in longitude and 10° in latitude. Tracked CMS were characterized by several variables. The SSH amplitude at each time step along a CMS trajectory was defined to be the difference between the SSH extremum in the CMS interior and the SSH value along the perimeter, delineated as the outermost closed contour of SSH that defined a compact structure. The horizontal speed-based radius scale of each feature, L_s_, was defined to be the radius of a circle with area equal to that enclosed by the SSH contour around which the average geostrophic speed was maximum within the feature’s interior, defined as the region inside the outermost closed SSH contour.

In the Gulf Stream region, eddies and meanders can be differentiated based on their direction of propagation: meanders generally propagate eastward while isolated eddies generally propagate westward^32,33^. In this study, we followed Gaube and McGillicuddy^34^ and defined individual CMS as being either an eddy or meander using the following criteria: if the net zonal displacement of a feature was westward, it was defined as an eddy, whereas if it was eastward, it was defined as a meander. Because some Gulf Stream meanders become eddies, we defined transformation of a meander into an eddy after 20 consecutive days of westward propagation. Likewise, an isolated westward propagating eddy can be reabsorbed by the Gulf Stream, becoming a meander. We allowed eddies to become meanders after 20 consecutive days of eastward propagation.

Gulf Stream meanders were identified as described above, with the additional constraint that they propagated in the Gulf Stream region defined by an envelope with northern and southern boundary 2° degrees north and 3° degrees south of the north wall of the Gulf Steam, defined as the average location of the 15° isotherm at 200 m estimated from World Ocean Atlas 2005 climatology^35^.

### Tags and Data Processing

Observations of the movement and behavior of the two mature female white sharks were collected by satellite tags deployed on the two individuals. The largest shark (WS12–17 in Skomal *et al*.^14^) was tagged on 17 September, 2012 off the coast of Cape Cod (USA) and weighed approximately 1,570 kg with a total length of 4.9 m at the time of tagging. The second shark (WS13–01 in Skomal *et al*.^14^) was tagged on 3 March, 2013 off of Jacksonville Florida (USA) weighing an estimated 900 kg and measuring 4.4 m in total length at tagging. No other individuals in Skomal *et al*.^14^ met the appropriate data quality and maturity criteria for inclusion in this study.

Both sharks were equipped with ‘Smart’ Position or Temperature Transmitting (SPOT) tags (Wildlife Computers, Inc.) mounted to the leading edge of the first dorsal fin allowing for high-resolution position estimates while the sharks swim at the surface. The SPOT tags were programmed to transmit location to the Argos satellite constellation while at the surface at a rate of up to 250 times per day. Locations of SPOT-tagged individuals were post-processed by Collecte Localisation Satellites (CLS) using a Kalman filter algorithm and were assigned error flags called location classes: LC 3, <250 m; LC 2, 250–500 m; LC 1, 500–1500 m; LC 0, >1500 m for classes 3, 2, 1, 0, respectively. Additional classes A, B represent positions derived from less than 4 satellite messages which result in no estimates of spatial accuracy from CLS; however, recent work on several marine species and platforms by Lopez *et al*.^36^ suggests error for A, B classes is order 1–10 km (max error <20 km). Location class Z positions are considered invalid and were removed from further analysis^37^. The average position error for the SPOT location estimates with the quality flags described above are generally less than 5 km^38^.

The second tagged shark was also equipped with a pop-up satellite archival transmitting (PSAT) tag (miniPAT tag; Wildlife Computers, Inc.) that recorded depth and temperature every 5 seconds throughout the tag deployment. Depth measurements were recorded at a resolution of 0.5 m with an estimated accuracy of 1% of the reading, and temperature was recorded at a resolution of 0.05 °C with an estimated accuracy of ±0.1 °C. These data were summarized onboard the tag for transmission through the Argos satellite network. Depth measurements were transmitted at 5-minute intervals, and this dive data are analyzed throughout the manuscript and a subset is shown in Fig. 3. The miniPAT tag was attached to the dorsal musculature lateral to the first dorsal fin near the midline with a small titanium dart and a stainless steel cable. The miniPAT tag was programmed for a 6-month deployment, after which the tag detached and floated to the surface, transmitting its data via the Argos satellite constellation.

The miniPAT temperature and depth measurements were georeferenced by linearly interpolating the SPOT tag positions to match the timing of miniPAT observations, allowing for the collocation of depth and temperature measurements to the interiors of CMSs.

To investigate the use of CMSs by white sharks, individual SPOT locations were collocated in space and time to the closest CMS. Our interest in this study was the use of eddies by white sharks. As such, we excluded all SPOT tag locations that occurred in regions where the water depth was less than 1000 m, which we used to define the edge of the region influenced by dynamics occurring on the continental shelf and slope.

Individual dive profiles were computed from the 5-min miniPAT data and defined as contiguous dives starting within 20 m of the surface and proceeding to a minimum depth of 200 m. In total, 429 individual dives were identified in the 181-day data record.

### Analysis of diel patterns in diving behavior

To examine diel patterns in the PSAT-tagged shark’s diving behavior, the observations were separated into day and night, where the day is defined here as the period starting one hour before local sunrise and ending one hour after sunset. We chose this definition based on acoustic observations collected by P. Gaube in the Northwest Atlantic that suggest mesopelagic prey are able to detect the light present at dawn and dusk.

### Definition of Eddy Subregions

To investigate if white sharks are more likely to be associated with the core, interior or periphery of cyclonic or anticyclonic eddies, we defined eddy subregions by the normalized distance ${\rm{r}}$ from the eddy SLA extremum (Supplementary Fig. 4). The eddy inner-core was defined as ${\rm{r}}\le {{\rm{L}}}_{{\rm{s}}}/2$. The outer core was defined as ${{\rm{L}}}_{{\rm{s}}}/2 < r\le {{\rm{L}}}_{{\rm{s}}}$ and the eddy interior was defined to include both the inner and outer core $({\rm{r}}\le {L}_{S})$. The eddy periphery was defined as ${{\rm{L}}}_{{\rm{s}}} < {\rm{r}}\le 2{{\rm{L}}}_{{\rm{s}}}$, and the area outside of an eddy was defined as ${\rm{r}} > 2{{\rm{L}}}_{{\rm{s}}}$.

### Identification of Geographic Subregions

Analysis of animations showing how the PSAT-tagged white shark interacted with CMS revealed substantial differences in diving behavior in the Sargasso Sea and the Gulf Stream region. The SSH amplitude of CMS also varied considerably between those two regions (*e*.*g*., Fig. 1). As such, the analysis of diving behavior was conducted separately in two sub-regions of the North Atlantic: an open-ocean region with bounds at 25°N, 79°W and 33°N, 62°W, and a Gulf Stream region, with bounds at 33°N, 77°W and 41°N, 55°W (Fig. 1). The white sharks also traveled into the region east of the Gulf Stream region (Fig. 1), where animations of white shark movement and mesoscale eddies revealed that they did not appear to spend considerable time in eddies, as indicated by the dominance of black points in this area in Fig. 1. As the focus of this study was to diagnose the use of mesoscale eddies by white sharks, we chose not to include tracks from this eastern region. Note that the radial histograms detailing white shark eddy use presented in Supplementary Fig. 1 include the observations in this eastern region, and a significant preference for the cores of anticyclones is still detected.

### Construction of Eddy Vertical Structure Composite Averages from Argo Float Profiles

Composite averages of the potential temperature structure of mesoscale eddies in the two regions investigated in this study were computed by collocating Argo float profiles to the interiors of mesoscale eddies. All quality-controlled Argo profiles within a distance of ±2° of the PSAT-tagged shark’s path were used to construct the composites. Although the miniPAT tag only remained attached for the 6-month time period 7-March, 2013 through 12-August, 2013, Argo profiles occurring during the 14-year time span 18-August, 2000 through 27-May, 2014 were used to construct the composite averages in order to take advantage of the ~14 year Argo data record.

Anomalies were computed for each temperature profile by subtracting the climatological temperature field from the World Ocean Atlas 2005^35^ interpolated in time and space to the Argo float profile location. All profiles within a normalized radial distance of 2L_s_ from the eddy SSH extremum were averaged separately for cyclones and anticyclones onto a uniform grid ranging from the surface to 1000 m at 10 m intervals in the vertical and from 0 to 2L_s_ with an interval of L_s_/10. The radial composite averages were mirrored along the y-axis and smoothed with a LOESS smoother with half-power cutoffs equivalent to a running-mean span of L_s_/4 in the horizontal and 40 m in the vertical.

## Electronic supplementary material

Supplementary Methods, Figures and Table
