# Supplementary material for: Mesoscale eddies influence the movements of mature female white sharks in the Gulf Stream and Sargasso Sea
Source: Sci Rep. 2018 May 9;8:7363. doi: 10.1038/s41598-018-25565-8 (PMC5943458; doi:10.1038/s41598-018-25565-8)
Supplement: Supplementary file 1 — Supplementary Methods, Figures and Table [file 41598_2018_25565_MOESM1_ESM.pdf]

Supplemental Information for:
Mesoscale eddies influence the movements of mature female white sharks in the Gulf Stream and Sargasso Sea

Peter Gaube, Camrin D. Braun, Gareth L. Lawson,
Dennis J. McGillicuddy Jr., Alice Della Penna, Gregory B. Skomal, Chris Fischer, and Simon R. Thorrold

Random shark trajectories

Random-walk trajectories originating at the tagging location of each of the white sharks analyzed in the manuscript were propagated forward in time at 1 day intervals for 6 months. The direction at each time step was selected from a uniform distribution of random directions ranging 0° and 359° at 1° increments. The propagation velocity was held constant as the average daily velocity of all the white shark position estimates (167 km cos(θ) per day), where θ is the latitude of the daily random-walk position.

The random trajectories were forced to remain within a box with vertices of 50°N, 76°W and 25°N, 25°W, and in regions where the water depth exceeds 1000 m. If a random-walk trajectory interacted with the boundary of this region, a new random direction was generated until the trajectory moved away from the boundary. An example of a single random-walk trajectory is shown in Fig. 5 in the extended data.

In total, 200 individual 6-month random walk trajectories were collocated to the interiors of CMS. There is no significant preference for the interiors of eddies of either polarity (Extended Data Figs. 5c and d). The results suggest that the observed pattern may indeed be a result of a preference for the inner-core of anticyclones.

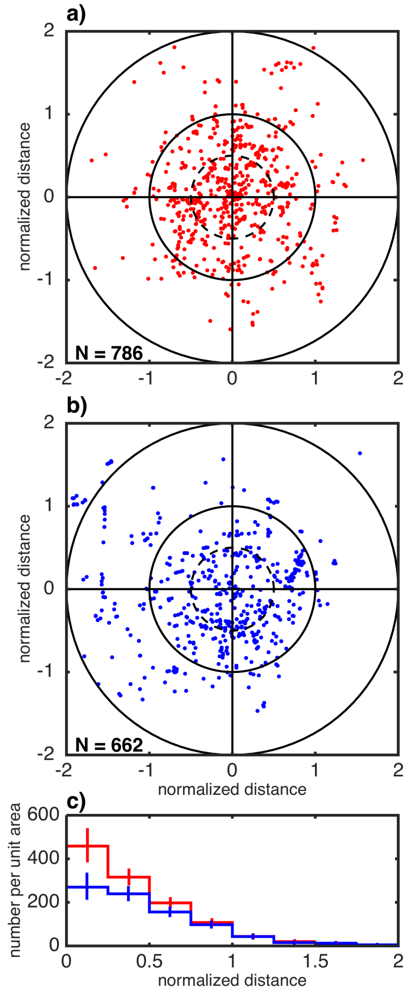

Supplementary Figure 1: Eddy-centric location maps of observed white shark SPOT tag position estimates (shown as magenta points in Fig 1 of the main text) inside the outermost closed contour of SLA defining (a) anticyclonic and (b) cyclonic eddies. The x and y axes of each panel have been scaled by the horizontal speed-based eddy radial scale L_s. (c) Histograms of the number SPOT locations per unit area of each radial annulus as a function of radial distance from the closest sea level anomaly extremum of mesoscale eddies.

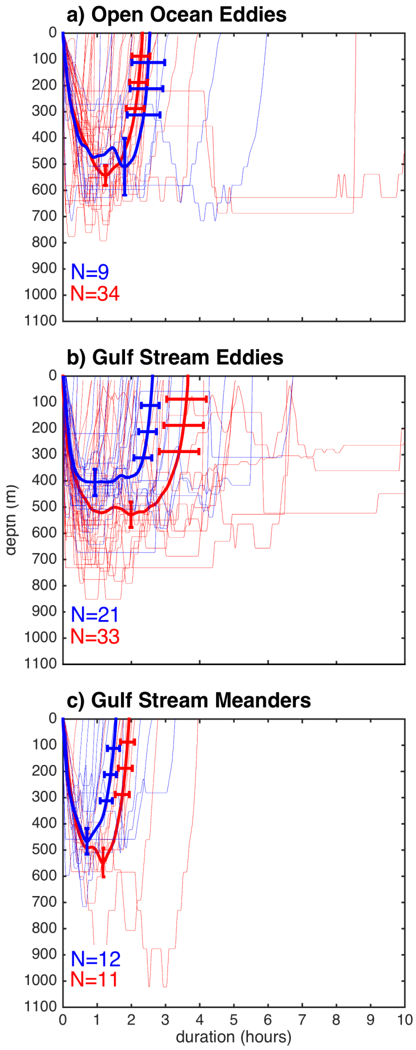

Supplementary Figure 2: Individual deep dive profiles (thin curves) and composite-averaged profiles (thick curves) of all dives occurring within the interiors of anticyclonic (red) and cyclonic (blue) eddies in (a) open-ocean eddies, (b) eddies in the Gulf Stream region and (c) Gulf Stream meanders. The 95% confidence interval of the composite-averaged dive depth at the deepest point in the composite- averaged dive profile are indicated by vertical lines. The 95% confidence interval of the composite-averaged dive duration are indicated by horizontal lines.

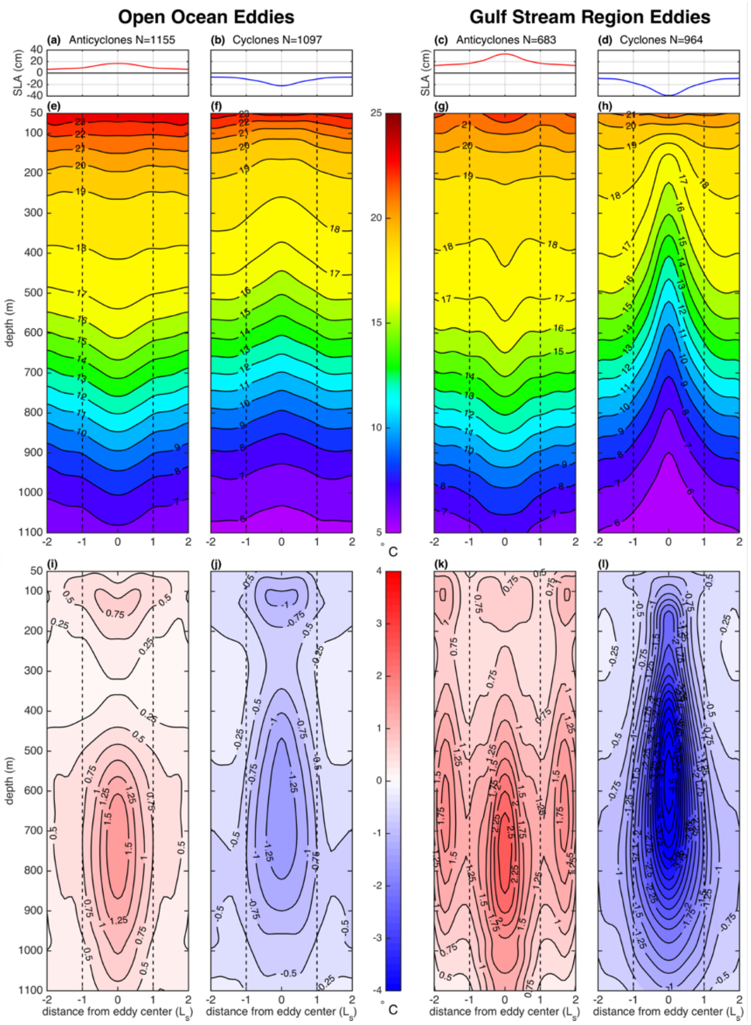

Supplementary Figure 3: Composite averages of open ocean anticyclones and cyclones are shown in the first and second column of figures, respectively. Composite average of anticyclones and cyclones in the Gulf Stream region are shown in columns 3 and 4, respectively. The top row of panels show radial composite averages of SLA at the location of the Argo float profiles used to construct the composite averages of potential temperature (middle row) and temperature anomaly (bottom row). The total number of quality-controlled Argo float profiles used in each composite is indicated by N at the top of each column.

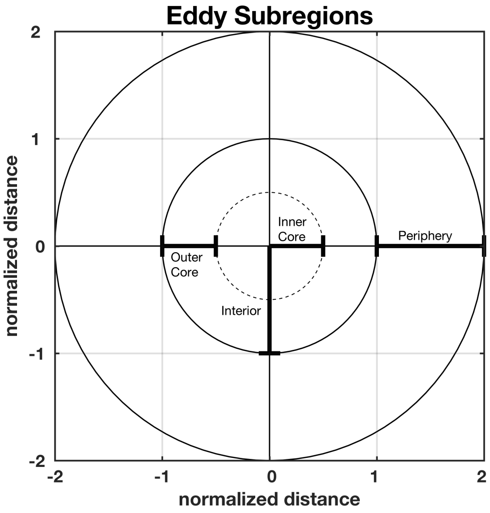

Supplementary Figure 4: Schematic representation of the various eddy subregion defined in the manuscript. The x and y axis represent distance from the eddy center normalized by the eddy radius scale L_s*.*

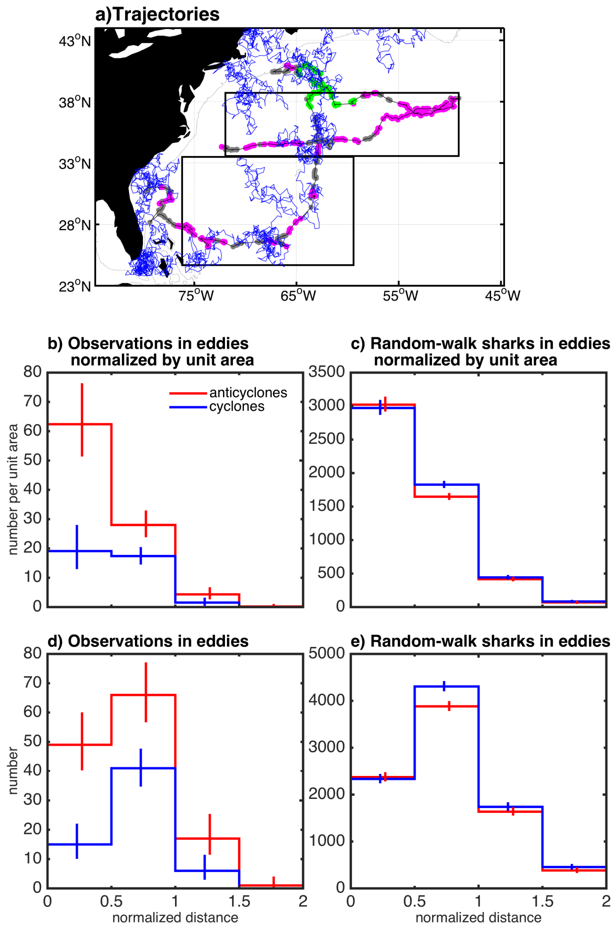

Supplementary Figure 5: The map in panel (a) shows the trajectory of the double-tagged white shark (grey line) overlaid with individual SPOT tag positions in eddies (magenta dots) Gulf Stream meanders (green dots) and outside of eddies or meanders (grey dots). This map was generated using the m_map toolbox ([https://www.eoas.ubc.ca/~rich/map.html)](https://www.eoas.ubc.ca/~rich/map.html) implemented in Matlab R2017a. The path of one representative random-walk trajectory is shown in blue. Histograms of the number of SPOT locations and random walk trajectories normalized by unit area of each radial annulus as a function of radial distance from the closest eddy sea level anomaly extremum are shown in panels (b) and (c), respectively. Histograms that were not normalized by area are shown in panels (d) and (e).

Supplementary Table 1: Diel diving behavior of the white shark in the two oceanographic regions shown in Fig. 1 expressed as percentage of total time spent above and below 200 m, referred to here as shallow and deep, respectively. Percentages are computed separately for observation inside cyclones and anticyclones.

	**DAY**		**NIGHT**	
	Deep	Shallow	Deep	Shallow
**GULF STREAM ANTICYCLONES**	50%	50%	35%	65%
**GULF STREAM CYCLONES**	40%	60%	29%	71%
**OPEN OCEAN ANTICYCLONES**	21%	79%	14%	86%
**OPEN OCEAN CYCLONES**	33%	67%	27%	73%
